# Do Countries Consistently Engage in Misinforming the International Community about Their Efforts to Combat Money Laundering? Evidence Using Benford’s Law

**DOI:** 10.1371/journal.pone.0169632

**Published:** 2017-01-25

**Authors:** Ioana Sorina Deleanu

**Affiliations:** Yale School of Law, Yale University, New Haven, Connecticut, United States of America; University of Texas at San Antonio, UNITED STATES

## Abstract

Indicators of compliance and efficiency in combatting money laundering, collected by EUROSTAT, are plagued with shortcomings. In this paper, I have carried out a forensic analysis on a 2003–2010 dataset of indicators of compliance and efficiency in combatting money laundering, that European Union member states self-reported to EUROSTAT, and on the basis of which, their efforts were evaluated. I used Benford’s law to detect any anomalous statistical patterns and found that statistical anomalies were also consistent with strategic manipulation. According to Benford’s law, if we pick a random sample of numbers representing natural processes, and look at the distribution of the first digits of these numbers, we see that, contrary to popular belief, digit 1 occurs most often, then digit 2, and so on, with digit 9 occurring in less than 5% of the sample. Without prior knowledge of Benford’s law, since people are not intuitively good at creating truly random numbers, deviations thereof can capture strategic alterations. In order to eliminate other sources of deviation, I have compared deviations in situations where incentives and opportunities for manipulation existed and in situations where they did not. While my results are not a conclusive proof of strategic manipulation, they signal that countries that faced incentives and opportunities to misinform the international community about their efforts to combat money laundering may have manipulated these indicators. Finally, my analysis points to the high potential for disruption that the manipulation of national statistics has, and calls for the acknowledgment that strategic manipulation can be an unintended consequence of the international community’s pressure on countries to put combatting money laundering on the top of their national agenda.

## 1. Introduction

The starting point of this paper is a collection of statistics on money laundering, published in 2010 by Eurostat. Eurostat stated that this data does not *“entirely comply with the stringent requirements of the European Statistics Code of Practice”* [1]. The Code of Practice contains 15 principles and is meant to reassure users that statistical authorities in Europe are impartial and produce trustworthy and objective statistics [2]. Compliance with these principles is determined by peer review, and non-compliance is not limited to only one principles. In particular, the lack of a mandate for data collection [3], the lack of adequate resources [4,5], and the lack of coherence and comparability across countries [1,6] have enjoyed support, as explanations for non-compliance. Alternatively, the possibility that statistics may (purposefully) not be accurately and reliably portray reality was dismissed, as a merely theoretical possibility [7]. Next to the nuisance associated with entertaining the likelihood of strategically manipulated statistics, the reason for this was that proving their manipulation is, in practice, very difficult. The mechanisms in place to check them are, often, not able to distinguish strategic statistics from inaccurate ones [7,8].

Standard economic theory proposed that successful misreporting of official statistics is not possible and certainly not sustainable [9,10]. Paradoxically, conventional wisdom tells us that countries do strategically manipulate their official statistics. For example, after a 2004 financial audit, Greece admitted to having lied about the extent of its budget deficit in order to join the Eurozone [11]. Following a 2006 leaked statement, Hungary’s prime minister admitted to having lied about the state of the economy, in order to win national elections [12]. This paradox is elucidated if we agree that manipulation is possible because detection is imperfect [13,14]. Consequently, when countries face serious conflicts of interest, which incentivises them to strategically manoeuvring their statistics, and when detection is imperfect, the honesty of national statistics should be questioned [8,15,16].

For this reasons, the setting in which the current global strategy to combat money laundering is played out, has been perfect for the study of the occurrence of strategically manoeuvred statistics. First, the Financial Action Task Force (FATF) has been standing at the core of the current global Anti-Money Laundering (AML) and Countering the Financing of Terrorism (CFT) strategy. This international watchdog has been mandated to create standards of conduct vis-à-vis money launderers and to enforce them worldwide [17]. The FATF and Moneyval (an associate organization of the FATF that has been organizing evaluations of countries that are not members of the FATF Group [18]) have periodically evaluated countries’ compliance with international standards, threatening to blacklist non-compliers. Hereafter, the term ‘international evaluations’ captures both the Mutual Evaluations Reports (MER) and the Follow-Up Reports (FUR) published by the FATF or Moneyval. Blacklisted countries are subject to economic embargo from the United States and their trade partners. This gives countries an incentive to comply with the FATF recommendations. Nevertheless, many elites did not view the strategy of the FATF as necessary for an immediate improvement in the fight against money laundering [19,20]. Moreover, aside considerations of efficiency, the literature [7,21] alluded to the conflict of interest entailed by the loss of *“high net worth individuals”*, of *“commissions”* for maintaining financial opacity and of other *“expected national benefits”* that fighting money laundering would entail. Despite this, in order to avoid blacklisting, countries have had to implement significant institutional changes in a speedy manner [22]. Moreover, these changes have had to reflect in the official statistics on compliance and efficiency in combatting money laundering, in time for the international evaluations. Nonetheless, opportunities for strategic manipulation presented themselves, as *“the assessors’ judgment is heavily dependent upon the quality and quantity of the information that the entities and authorities are willing to share*” [23].

Despite the frequent use of the term, there is ambiguity with respect to what constitutes strategic manipulation of statistics. Statistics can be completely invented, marginally altered, or omitted [24], in order to aid the country’s overall and relative ratings. Some of these interventions may leave a trace in the form of numerical anomalies, which can be picked-up using statistical tools (*e*.*g*. Benford’s law). Nevertheless, the presence of numerical anomalies does not prove strategic manipulation, let alone reveal the identity of the culprit. The reason thereof, is that, statistical records involve complex processes where errors and unforeseen events are ubiquitous. Having said this, I was only interested in pointing out numerical anomalies that correlated with countries’ incentive to misrepresent their statistics. Against this backdrop, this paper asked the question: *did countries consistently engage in misinforming the international community about their efforts to combat money laundering when faced with the incentives and with the opportunities to do so*?

The paper unfolds with an overview of the recent developments in the literature on cheating and strategic manipulation (Section 2). Having positioned the paper in this literature, in Section 3, I describe the data and the methodology used to assess numerical anomalies. In Section 4, I apply the forensic test and compare anomalies across a range of incentives and opportunities for strategic manipulation. Section 5 concludes with a discussion on the integrity of European statistics on money laundering and recommends different research paths.

## 2. Literature overview

A fundamental assumption many economic models made, is that economic agents are rational and have knowledge of the true state of the economy [10]. Under this assumption, governments that cheat would be unmasked instantly [9]. Knowing that they will certainly face detection and punishment, according to Becker’s simple model of rational crime [25], government officials would not even try to cheat. However, when fundamental uncertainty exists (*e*.*g*. when detection is imperfect), the literature showed that relying on an expert’s possible ‘cheap talk’ may be an equilibrium [13,14,26]. Moreover, behavioural economist Dan Ariely and colleagues have argued that cheating is not a result of a cost and benefit analysis but a product of people’s lack of accuracy in self-assessment [27–29]. Consequently, cheating is the outcome of mitigating a good self-perception with benefiting from cheating. Consequently, the lack of self-monitoring, peer-monitoring or transparency are found to give opportunities for cheating [30], while the presence of a conflict of interest gives the incentive to cheat.

In recent years, a growing body of academic works has explored the extent to which cheating occurs in other fields where opportunities (*i*.*e*. where peer monitoring is imperfect or where transparency is low) and incentives for cheating exist. Shifting from laboratory experiments to the analysis of statistical records, this literature used a deeply non-intuitive, yet fascinatingly simple law of Benford, which formalized the frequency distribution of the first digits of naturally generated series of observations. Additionally, this literature leaned onto two crucial assumptions: people do a poor job in replicating known data-generating processes [31] and Benford’s law is not widely known by those constructing the data under investigation. With this in mind, Benford’s law became a good tool to investigate human driven data unreliability. Consequently, the scope for the application of Benford’s law was initially limited to the settings prosaically associated with strategic misreporting: tax filings [32], political campaign financing and election results [33,34], and survey results [35,36]. Stimulated by the encouraging results of these early exploratory works, a new wave of explanatory works used Benford’s law to test theories on cheating in settings less often associated with strategic misreporting: balances of payments [8], budget deficit statistics [15,37], academic publications [38,39] and macroeconomic statistics [16,40,41].

To present date, only few authors have investigated the allegation that countries strategically manipulate their official statistics. And, while not all studies have found evidence of strategic manipulation, they have confirmed the applicability of the law for investigating the quality of national statistics. The main difficulties faced by these investigations have been–on the one hand–putting together a dataset that is sufficiently robust for the application of Benford’s law [8], and–on the other hand–identifying the culprit decision maker who manipulated the statistics. For example, Nye and Moul used Benford’s law to test the argument [42] that Penn World Tables macroeconomic indicators of less developed countries are less reliable. Due to data scarcity, they bundled together OECD data and African data and compared the divergence from Benford’s law of these two groups. As expected, OECD macroeconomic indicators deviated less, but it remained unclear where and how manipulation occurred [40,41]. Similarly, Michalski and Stoltz used Benford’s law to test the hypothesis that “*a country may want to hide its true state of the world to prevent capital outflows or attract inflows”* [8]. For this purpose, they grouped countries according to their exchange rate regimes, foreign asset and current account balance positions, and according to their vulnerability to capital flow reversals and showed that, indeed, countries that had extra financial incentives to cheat, also had balances of payments with significantly higher deviations from Benford’s law. Likewise, Rauch and colleagues took the view that *“like firms*, *governments might try to make their economic situation seem better”* [15]. They used Benford’s law to measure country specific deviation of macroeconomic data relevant in determining whether European Member States comply with the deficit criteria set by the EU Stability and Growth Pact. They then, ranked countries according to the extent to which they diverged, arguing that “*the position of each individual country in this ranking helps to determine in which order and to what extent further auditing procedures should be carried out”* [15]. Their analysis showed that Greece–a country plagued with allegations of deficit data falsifications–was the first to need an audit. Later, Rauch and colleagues used Benford’s law to show that the deviations of the statistics on social security were considerably smaller than deviations of the statistics on deficits across the EU. They concluded that *“European governments behave in accordance with the incentives*, *i*.*e*. *while the quality of the social security statistics appears to be higher*, *there is a widespread tendency to report incorrect deficit data”* [37].

Finally, the literature warned that crime data is prone to misinterpretation, as it reflects the size of crime in an area, the degree to which law enforcement is willing and able to handle it, and the degree of interest in fighting the crime [43,44]. Over time, odd crime and crime-fighting statistics have raised questions, yet doubts were often put to rest with two explanations: European law enforcement agencies lack the capacity and the resources to keep more reliable statistics [45], and developing countries experience this problem even more broadly [36,46]. Alternatively, the possibility that political and economic pressures may underlie official statistics have been far fewer and much more disputed. For example, in the early 1930s, the US National Commission on Law Observance and Enforcement issued a report on Criminal Statistics where it argued that police data has a high probability to be falsified due to laxer legislation on police procedure and due to self-interest pressures. *“The significant fact that cities are beginning to use these reports in order to advertise their freedom from crime as compared with other municipalities suggests at once a difficulty under which the voluntary system of gathering police statistics for national purposes must labor”* [47]. Shortly after, the report was criticized by Davies who argued that *“cases of deliberate falsification are practically unknown”* [48]. More recently, brought to light through the movie Freakonomics [49], were the controversial allegations that Japanese homicides rates were falsified. In his memoires, Hiromasa Saikawa, a former member of the Tokyo Metropolitan Police, reportedly unveiled that police officers were obsessed with having perfect statistics, in order to get promoted [50]. Finally, in the particular care of statistics on money laundering, Masciandaro and Portolano argued for not excluding *“the possibility that some […] countries are not presently included in the FATF monitoring action*, *[…] because they are highly effective in bringing their formal rules in line with international precepts*, *while in their deeds they remain lax […]*. *This implies a constant effort of […] the FATF*, *to update the criteria and monitor countries”* [7]. However, the authors have not been able to empirically support their allegation. Finally, while efforts to empirically prove fraudulent manipulation of ‘paper reality’ by means of field experiments [51], investigative journalism (*e*.*g*. Luxembourg leak [52], Panama papers [53]) and non-parametric estimations (*e*.*g*. some authors [8,15,16,40] used Benford’s law to investigate whether countries strategically modify specific macroeconomic statistics) have since surged, the allegation that countries may strategically manipulate their statistics on compliance and efficiency in combatting money laundering has not yet been empirically explored.

## 3. Materials and Methods

### 3.1. A sample that would be prone to misreporting

The first challenge of this paper was to compile a dataset of statistics that could have been manipulated by countries facing international pressures to fight money laundering in order to avoid being blacklisted. My sample consists of official statistics on compliance and efficiency in combatting money laundering pertaining to 27 EU Member States. Although a comparison with the US, Australia and other EEA members was possible and might be warranted, my sample represents a relatively more heterogeneous set of AML/CFT regulatory regimes and actions [54], as well as a set of socio-economic institutions on a path of converge [55]. Focusing exclusively on the EU had the additional advantage of being able to make use of Eurostat’s official statistics on compliance and efficiency in combatting money laundering. Statistics that were provided by the Member States were then checked for consistency by Eurostat, and therefore, added to a more consistent and reliable dataset. Eurostat presented these statistics in two different reports. The 2010 Eurostat report contained data from 2003–2008 and the 2013 Eurostat report contained data from 2005 to 2010. When merging the two datasets, I came across several data misfits, and I have consistently chosen data published in the latter report, assuming it contained fewer mistakes. Finally, I complemented the Eurostat database (where data was missing) with data from official annual reports and from international evaluations. I considered this a helpful and innocuous fix, despite the fact that, in fact, I may have overlooked fake statistics that were later passed as mistakes or omissions, or have overlooked manipulation by omission.

In the period 2003–2010, each Member State underwent an international evaluation–conducted by FATF or Moneyval representatives–as part of the 3^rd^ Round of Mutual Evaluations. The FATF standards have been adjusted with each round, so it is fair to say that the benchmark for comparison and progress measurement is the previous evaluation. Mutual evaluations are a form of peer-monitoring–whereby a team of foreign mixed-nationality evaluators spends a minimum of 7–8 days in a country, conducting interviews, observing the current state of affairs of the AML/CFT system, and inquiring into past statistics [56]. The evaluations are meant to check the extent to which countries complied with the above-mentioned recommendations. As a result, countries were awarded one of four qualifications (Table 1). The qualifications ‘compliant’ and ‘largely compliant’ were considered good. If a country received a ‘partially compliant’, there were serious remedies that the country needed to undertake to signal compliance with the FATF standards [57–59]. Finally, ‘non-compliant’ was the worse qualification, that would lead to significant international pressure being put on the country to address it, and that may ultimately lead to economic sanctions [60]. Of the 40+9 recommendations published by the FATF, only a few required numerical support. Consequently, my dataset contains statistics recorded under the FATF’s recommendations 13, 16, 27, 31, 32 and special recommendation IX. Table 1 reports on the qualifications countries received hereon.

**Table 1:** Year when the MER was published and the evaluation results on Recommendations 13, 16, 27, 31 and 32 and on Special Recommendation IX by EU Member State.

Country ISO code	Publication MER	R13	R16	R27	R31	R32	SR IX
**AT**	2009	PC	PC	C	C	PC	PC
**BE**	2005	LC	LC	C	LC	LC	NC
**BG**	2008	PC	PC	LC	C	PC	PC
**CY**	2006	C	PC	LC	C	PC	LC
**CZ**	2007	LC	PC	C	PC	LC	LC
**DE**	2010	PC	NC	LC	LC	PC	LC
**DK**	2006	PC	PC	C	LC	PC	PC
**EE**	2008	LC	PC	C	LC	LC	PC
**EL**	2007	PC	NC	LC	PC	NC	NC
**ES**	2006	LC	PC	LC	LC	PC	LC
**FI**	2007	LC	PC	LC	LC	PC	PC
**FR**	2011	PC	PC	LC	LC	PC	LC
**HU**	2005	PC	PC	LC	C	LC	PC
**IE**	2006	C	PC	C	LC	PC	PC
**IT**	2006	PC	NC	C	LC	LC	C
**LT**	2006	PC	PC	PC	LC	PC	PC
**LU**	2010	NC	NC	PC	PC	PC	NC
**LV**	2006	LC	NC	C	LC	LC	NC
**MT**	2007	PC	PC	LC	C	LC	LC
**NL**	2011	LC	PC	C	LC	LC	LC
**PL**	2006	PC	NC	PC	PC	PC	LC
**PT**	2006	LC	PC	LC	LC	PC	LC
**RO**	2008	PC	NC	LC	LC	LC	PC
**SE**	2006	PC	PC	LC	LC	PC	NC
**SK**	2006	PC	NC	LC	PC	PC	PC
**SL**	2005	PC	PC	PC	C	LC	C
**UK**	2007	C	LC	C	C	LC	LC

**Notes.** In accordance with the 3^rd^ Round of Mutual Evaluations, published by the FATF and Moneyval. C, LC, PC, NC mark compliant, largely compliant, partially compliant, and non-compliant.

Table 1 shows that Member States were not evaluated in the same year. If conducted at the beginning of the year, evaluators would only be able to take statistics of the previous year into account. In general, the report on the evaluation was published 6 to 9 months after the evaluation took place (exceptions were the Czech Republic and Malta, which took more than 1 year before the report was published) [56]. For indexing purposes, I considered the evaluation to be published one year after the evaluation takes place.

Furthermore, the FATF’s *“Third Round of AML/CFT Mutual Evaluations -Process and Procedures”* guidelines proposed that countries should present a Follow-Up Report (FUR) two years after the publishing of the MER. Presenting the FUR in an FATF plenary session was comparable to publishing the results of a self-evaluation that would be subject to peer-monitoring–thereby increasing transparency. The country presenting had to report on the progress it had made since the mutual evaluation, particularly with respect to those recommendations where it received a partially compliant or a non-compliant qualification. Countries that had no such qualifications would not need to present a FUR, but no such example was present in my sample. In a FUR, great emphasis was placed on statistics, especially if they were found to be lacking in the mutual evaluation [56]. FATF plenary meetings took place three times a year, in February/March, June/July or October/November, respectively. Countries had to send their FURs to the plenary in advance, as to allow for discussion [61]. Consequently, the FUR usually contained statistics of the previous year and even earlier statistics.

In conclusion, countries are assumed to self-monitor their AML/CFT system, as well as the quality of their own statistics. In contrast, peer monitoring (*i*.*e*. mutual evaluations) exists in some years, and in others, it does not, and FUR presentations can be thought of as peer monitoring or increases in transparency. Finally, peer monitoring and transparency add to self-monitoring.

### 3.2. A sample that allows for misreporting to be detected with Benford’s law

In 1881 astronomer and mathematician, Simon Newcomb, wrote in an article in the American Journal of Mathematics where he noted that first digits do not appear with equal frequency in large sets of ‘real-world numbers’–*i*.*e*. numbers that reflect natural processes. He observed that the leading digits *d* ∈ {1,…,9} of naturally generated series of observations occur with probability $P(d)=\log_{10}(d+1)-\log_{10}(d)=\log_{10}\left(1+\frac{1}{d}\right)$ [62] (see Table 2 for the numerical approximation). His observation went against the popular belief that in large datasets, the numbers 1 to 9 should appear with equal probabilities as leading digits.

**Table 2:** Numerical expression of Benford’s law: frequency distribution of leading digits *d* ∈ (1,9).

*d*	1	2	3	4	5	6	7	8	9
***P*(*d*)**	30.1%	17.6%	12.5%	9.7%	7.9%	6.7%	5.8%	5.1%	4.6%

In 1938, American physicist Frank Benford tested the observation of Newcomb, on an own collection of ‘real world numbers’ among which population figures, baseball statistics and numbers appearing in Reader’s Digest articles [63]. Benford found that he could empirically confirm the predictions of Newcomb very well, and so, although discovered by Newcomb, the law took the name of Benford who popularized it. A century after it was first discovered, Hill proved the law–namely that “*if probability distributions are selected at random*, *and random samples are taken from each of these distributions in any way so that the overall process is scale (base) neutral*, *then the significant-digits frequencies of the combined sample will converge to the logarithmic distribution”* [64]. Gauvrit and Delahave reformulated the necessary conditions and argued that *“scatter and regularity are […] sufficient conditions for Benfordness”* [65]. Similarly, Janvresse and De La Rue proposed that mixtures of uniform distributions lead to samples whose leading digits conform to Benford’s law [66].

Consequently, the second challenge that this paper faced was to ensure that this dataset complies with these necessary and sufficient conditions for Benford’s law to apply. Failure to do so would have otherwise led to the a-priory rejection of conformance to Benford’s law [67]. I therefore dropped the statistics on compliance and efficiency in combatting money laundering that were base invariant (*e*.*g*. number of staff dedicated to fighting money laundering in the Financial Intelligence Unit (FIU), Police, Judiciary) and that were interval-bound (*e*.*g*. the percentages of suspicion reports coming from credit institutes, sent to law enforcement, investigated by law enforcement).

Additionally, addressing the scatter condition, my dataset reflects different processes susceptible to exponential growth, measured across 27 EU Member States over a period of eight years (from 2003 to 2010). My dataset, therefore, contains the number of suspicion reports put forward by different groups of reporting entities, the number of suspicion reports analysed by law enforcement, the number of criminal ML investigations, prosecutions and convictions, and the number of correct and incorrect cash declarations filled in at border crossings, and the amounts of cash moved across borders (see Table B in S1 File). Furthermore, aggregating data from different processes over several countries increased the likelihood that the dataset conforms to Benford’s law [8]. This is particularly relevant as missing data for some countries in my dataset, may not make these time series particularly representative for economic processes with exponential growth rates (see Table A in S1 File). Moreover, the processes under investigation were various enough to ensure that data “covers several orders of magnitude” [68] even when considering only national samples. Finally, since Benford’s law applies, in theory, to continuous random variables, I employed Monte Carlo simulations in testing whether the data are conforming to Benford’s distribution [69].

Nevertheless, there are several limitation with respect to what Benford’s law can pick-up in terms of numerical anomalies. First, small-scale data alterations (non-first digit) are not picked-up. Second, Finland, Germany and Spain published almost twice as many statistics on compliance and efficiency in combatting money laundering than Greece and Poland (see Table A in S1 File). Missing data may affect the probability distribution of the first digits. Finally, data limitations require an analysis at an aggregate level. This implies that Benford’s law is less likely to be informative with respect to the source of the alleged manipulation. Certainly, it cannot confirm beyond doubt that manipulation occurred.

### 3.3. Measuring a sample’s goodness of fit to Benford’s frequency distribution

I used several measures of distance between the empirical data and Benford’s distribution, each accounting for different characteristics that the data and the samples may have. Seeing how there are arguments to be made on more of these characteristics, I measured deviation from Benford’s law with all available tools, and argued that, unless these characteristics played a major role, all these measures should have converged in revealing statistically significant deviances. The null hypothesis is that the first digits of the statistics on compliance and efficiency in combatting money laundering are drawn from Benford’s distribution. I considered that the null hypothesis was rejected if the *χ*^2^ test, the Kolmogorov-Smirnov and the modified Kuiper test, all, rejected it at 1%, 5% or 10% level.

The *χ*^2^ goodness-of-fit test compares the empirical frequencies of the data under analysis $\hat{\theta_{j}}$ with the frequencies of data following the distribution of Benford θ_j_,

$$\chi^{2}=N\sum_{j=1}^{9}\frac{{{(\theta }_{j}-\hat{\theta_{j}})}^{2}}{\theta_{j}}$$

Where *N* denotes the total number of observations. Note that *j* ≠ *i* as one indexes countries and the other indexes samples, some of which containing cross-country data. However, the *χ*^2^ test has a particular drawback in its sensitivity to small samples [33]. Furthermore, given the limited degrees of freedom, the test is powerful only for samples where *N* > 110 [8]. Since some of my sub-samples were smaller, I made use of the multinomial-goodness-of-fit test, which samples *n* units with replacement from a population of *k* elements of the Benford’s law distribution [69]. The number of replications for the sampling is 10,000. The exact p-value is equal to the fraction of replications in which the test statistic is at least as large as in the data under analysis.

The Kolmogorov-Smirnov test has higher power than the *χ*^2^ test and is particularly appropriate for data having a natural order, which digits have. The test captures the highest absolute difference between the Benford’s law and my observed, distribution function. Since the test is not meant for discrete data, I first transformed the data using the Monte Carlo method and then ran the Kolmogorov-Smirnov test [69]. I consider *KS* = *max*_1≤*j*≤9_ [|*H*(*j*) − *F*(*j*)|], with $H(j)=\frac{1}{n}\sum_{i=1}^{j}h_{i}\;and\;F(j)=\frac{1}{n}\sum_{i=1}^{j}f_{i}$,

Where *h*_*i*_ and *f*_*i*_ are the observed and, respectively, Benford’s counts for digit *j* [70].

The Kuiper test is a modified Kolmogorov-Smirnov goodness-of-fit test that recognizes both the ordinality and the circularity of the data on leading digits. The Kuiper test sums the maximum of the positive and of the negative deviations from Benford’s law.

$$V_{N}=\underset{j}{\max}\;[|H(j)-F(j)|]+\underset{j}{\min}\;[|F(j)-H(j)|]$$

I adjusted the Kuiper test using Stephens’ correction factor ${V_{N}}^{*}=V_{N}[\sqrt{N}+0.155+0.24\frac{1}{\sqrt{N}}]$ [71] and applied asymptotically valid critical values (approximately 1.19 at 10%, 1.32 at 5%, and 1.58 at 1%) [72].

In addition to the statistical tests, I included two measures of distance between the data under observation and Benford’s distribution. These fall outside the hypothesis testing framework and are insensitive to the sample size (24, 60). I calculated the Chebyshev distance $m=\max_{i=1,\ldots ,9}\left\{\right|{(\theta }_{j}-\hat{\theta_{j}}|\}$ [73] and the Euclidean distance between the two distributions, which I divided by the maximum possible distance (which would occur when all numbers begin with an FSD of 9) so that the value is bounded between zero and one: $d^{*}=\frac{\sqrt{\sum_{j=1}^{9}{{(\theta }_{j}-\hat{\theta_{j}})}^{2}}}{1.0363}$.

### 3.4. Institutional limitations, incentives and opportunities for strategic manipulation

The third challenge of this paper was to place the assessment of numerical anomalies in the institutional context in which the indicators of compliance and efficiency in combatting money laundering were produced. Statistic gathering is a complex processes, and serves two purposes: (1) the identification and description of trends and typologies concerning money laundering [74] and (2) the review of the effectiveness of the national AML/CFT strategy [75]. As pivotal to the effectiveness of the strategy, the FIU played an important role in the collection and dissemination of statistics on money laundering, often in partnership with representatives of the law enforcement authorities. Depending on whether these statistics were used to meter its performance [60], it may also have had a stake. Consequently, the explanations offered for why these statistics do not comply with the European Statistics Code of Practice, should have some representation in the FIU. After all, a well-staffed FIU, with adequate budget, and with a mandate to collect data cannot be expected to provide low quality statistics. And, while an analysis of the staffing and budgeting of the other national agencies responsible with the collection of statistics on money laundering is warranted, such data is much less available [1,20,76]. Finally, I do not wish to imply that the FIU acted as the national decision maker, judging whether to manipulate statistics or not.

## 4. Results

In all analyses, I have limited the discussion to the results yielded by sufficiently large samples that spanned across sufficient degrees of magnitude (*e*.*g*. *N* > 110 and *Scatter* > 4 in Table A and Table B in S1 File). A first check of conformance reveals that my entire dataset rejects Benford’s law at 1% (χ^2, K-S and Kuiper tests). This implies that some anomalies exist. In order to get a better grasp of what constitutes an anomaly, consider a dataset *H* containing the heights (measured in centimetres) of all adult men in the EU, and a dataset F containing the amounts of Euro contained in accounts frozen on suspicion of money laundering in the EU. Dataset *H* does not obey Benford’s law because, to date, adult men do no measure less than 50cm or more than 2.9m in height [77]. This entails that *P*_*H*_(3) = *P*_*H*_(4) = 0 and *P*_*H*_(1) → 1. Despite non-conformance to Benford’s law, this is not an anomaly. The same cannot be said if we were to observe the same distribution of first digits in dataset *F*. A dataset contains anomalies when it does not conform to Benford’s law, while it theoretically should.

Anecdotal evidence suggests that numerical anomalies are due to the novelty of the process of data gathering, budget, and capacity constraints, issues of complexity, and lack of mandate. For this to hold, statistics on money laundering should deviate more from Benford’s law when FIUs have fewer staff (see Table C in S1 File) and when more statistics are compiled (see Table A in S1 File). Moreover, deviations should decrease over time, with the overall expansion of the FIU’s mandate and with routine. Data on FIU budgets and law enforcement budgets is too scarce to draw any conclusion [20,76].

In order to test these hypotheses I aggregated, by year, all European statistics on money laundering. Table 3 reports on the yearly deviations of these statistics and shows that they did not decrease over time. Moreover, yearly deviations did not increase when more statistics were collected, and higher FIU staff capacity is not correlated with statistics deviating less from Benford’s law. Table 3 reports on two statistics: (1) the upper echelon measures the deviation from Benford’s law of all known statistics organized by year; (2) the lower echelon measures the deviation from Benford’s law of statistics pertaining to Member States whose yearly budgets are known. We assumed the latter echelon captures more accurately the relationship between the capacity of the FIU and the quality of indicators of compliance and efficiency in combatting money laundering. Note that since the statistics under analysis exclude staff numbers (see Section 3), correlations between deviations from Benford’s law and staff numbers are not inherently biased. Finally, countries with better-staffed FIUs publish more statistics, just not statistics with fewer anomalies.

**Table 3:** Assessment of conformity to Benford’s law, by year, across all Member States and across Member States for which data on the FIU staff is available.

Year	*N*	*χ*^2^	*K* − *S*	*V*_N_*	*χ*^2^/*N*	*m*	d*	*Scatter*	$\bar{Staff}$
**Assessment of conformity to Benford’s law, by year, across all Member States**									
2003	216	18.622**	0.055	0.9	0.078	0.06	0.1	4	20.44
2004	257	9.893	0.042	0.94	0.052	0.04	0.08	5	20.16
2005	297	5.005	0.036	0.68	0.048	0.04	0.05	5	21.43
2006	336	9.055	0.059*	1.4**	0.062	0.06	0.07	5	23.69
2007	364	20.185**	0.078***	1.52**	0.054	0.08	0.09	8	28.30
2008	562	14.495*	0.033	1.26*	0.058	0.02	0.04	10	32.95
2009	558	8.205	0.034	0.85	0.027	0.03	0.04	10	30.25
2010	533	13.672*	0.06**	1.77***	0.065	0.04	0.05	10	30.80
**Assessment of conformity to Benford’s law, by year, only across Member States for which the FIU staff is known**									
2003	153	21.501***	0.078	1.07	0.141	0.08	0.13	4	20.44
2004	190	11.386	0.052	0.84	0.060	0.05	0.1	4	20.16
2005	223	5.004	0.048	0.77	0.022	0.03	0.05	4	21.43
2006	268	8.899	0.062*	1.34**	0.033	0.05	0.07	4	23.69
2007	318	13.337*	0.054	1	0.042	0.05	0.07	8	28.30
2008	462	21.642***	0.058**	1.79***	0.047	0.03	0.06	10	32.95
2009	438	4.668	0.027	0.59	0.011	0.02	0.03	10	30.25
2010	424	13.327	0.065**	1.78***	0.031	0.04	0.06	10	30.80

**Notes.** Deviation is captured using the Kuiper test, the Kolmogorov-Smirnov test, the *χ*^2^ test (adjusted for sample size), the Euclidean distance (d*) and *m*. The columns ‘Scatter’ and ‘Staff’ represent the degrees of magnitude the sample passes through and the corresponding yearly average staff capacity of the FIUs for which data is available.

*p< .1

**p< .05

***p< .01.

Alternatively, the hypothesis of strategic manipulation states that peer monitoring and increased transparency reduce the perceived opportunity to engage in unethical behaviour [27,30], thereby reducing the chance for strategic manipulation of statistics to occur. Consequently, I expect that numerical anomalies were less numerous in the years of the mutual/ follow-up evaluations.

**Fig 1:**
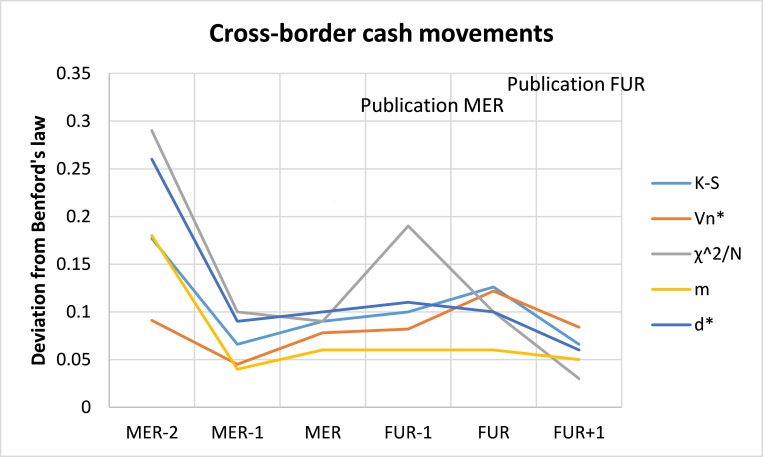
Timely assessment of conformity to Benford’s law – statistics on compliance and efficiency in combatting money laundering. While in accordance with Table 3, for visual purposes the measures $V_{N}^{*}$ has been divided by a factor 10.

In order to test this and given the limited data availability, I pooled the statistics on compliance and efficiency in combatting money laundering across the 27 EU Member States, according to their distance relative to the evaluation moment. For the same reason, I started three years before the mutual evaluation. Since evaluations did not all take place in the same year, comparison of deviations is less likely to have captured the effect of exogenous factors, such as, the financial crisis, changes in book keeping standards etc. Fig 1 plots a succession of two evaluation cycles. Evaluations took place at time MER and FUR, and the results thereof were published the year after at FUR-1 and at FUR+1. Statistical anomalies occurred least when peer monitoring or transparency increase, thereby offering some support to the strategic manipulation hypothesis.

Additionally, Table 4 reports on two measures that confirm the appropriateness of using Benford’s law to detect data irregularities, namely the sample size (*N* > 110), and the degree to which data is scattered (*i*.*e*. data passes through at least four degrees of magnitude). Once again, the staff capacity of the FIU and the number of collected statistics do not explain the occurrence of numerical anomalies.

**Table 4:** Assessment of conformity to Benford’s law before and after international evaluations.

Variables ^a^	*N*	*χ*^2^	*K* − *S*	*V*_N_*	*χ*^2^/*N*	*m*	d*	Scatter	$\bar{Staff}$
ML stats MER-3	159	14.09*	0.101**	1.38**	0.09	0.08	0.1	4	24
ML stats MER-2	264	11.08	0.044	0.75	0.04	0.02	0.04	9	25
ML stats MER-1	318	7.57	0.037	0.71	0.02	0.02	0.03	10	23
ML stats MER	349	14.74*	0.045	0.85	0.04	0.02	0.03	10	23
ML stats FUR-1	326	20.3***	0.095***	1.75***	0.06	0.05	0.06	10	22
ML stats FUR	374	6.2	0.055*	1.24*	0.02	0.03	0.03	9	31
ML stats FUR+1	409	16.28**	0.08***	1.87***	0.04	0.03	0.04	9	30

**Notes**.

^a^ ML stats MER-3, ML stats MER-2, ML stats MER-1 and ML stats MER aggregate statistics on compliance and efficiency in combatting money laundering that were published 3,2 and 1 year before and respectively during the year of the international evaluation. ML stats FUR-1 and ML stats FUR aggregate the statistics published the year before and respectively during the compilation of the FUR. ML stats FUR+1 aggregate statistics on compliance and efficiency in combatting money laundering that were published the year the FUR was published and discussed in the plenary. Staff represents the average staff capacity of FIUs whose statistics are evaluated

*p< .1

**p< .05

***p< .01.

The presence of a conflict of interest implies that strategic manipulation has a positive benefit (large or small) [27]. Consequently, badly evaluated countries have a conflict of interest after the evaluation, whereas positively evaluated countries do not. Therefore, did countries that were badly evaluated have more numerical anomalies?

In order to test this, I grouped countries into four categories ranked 1 (best conforming) to 4 (least conforming) according to their evaluations on recommendations 13, 16, 27, 31 and 32. I selected first, only statistics recorded after the mutual evaluation took place, consequently aiming to capture the effect of the mutual evaluation on deviations in the second evaluation cycle. In Group 1, I aggregated statistics of countries that have been rated C or LC, and only on maximally one account PC on recommendations 13, 16, 27, 31 and 32. In Group 2, I pooled statistics of countries that performed less well, in the sense that their panoply of good evaluations was spoiled only on two accounts with a PC, or on one account with a NC. In Group 3, I aggregated statistics of countries that were being rated on three accounts with PC or on two accounts–once PC once NC. Finally, in Group 4, I pooled data of countries that were evaluated by the international community with PC or NC on most if not all accounts. Seeing how countries that performed best also had significantly higher numbers of staff, both before and after the MER (see Table C in S1 File), numerical anomalies could be explained by the eventual incentives to manipulate statistics, as well as by the lack of adequate resources. In order to disentangle the two effects, I compared deviations from Benford’s law across groups, before and after the mutual evaluation. I could do this, as the number of FIU staff employed is largely idiosyncratic [$pwcorr({\bar{Staff}}_{until},{\bar{Staff}}_{after})=0.96^{**}$].

Table 5 reports the divergence of these four groups from Benford’s law. After the mutual evaluation, the group that pooled statistics of the better-rated countries did not significantly deviate from Benford’s law, while the rest did. Group 1 also has the highest average of FIU staff members. Nevertheless, while staff ratios are relatively stable across the four groups of countries before and after the mutual evaluation, the deviation patterns are not. The hypothesis that FIU capacity matters for reducing numerical anomalies in statistics is only supported in the case of the lowest staffed FIUs (*i*.*e*. Group 4 in Table 5). Note however, that Benford’s law can only pick large numerical anomalies (that are present in the first digit) and therefore, it is possible that missing statistics or smaller alterations are overlooked.

**Table 5:** Assessment of conformity to Benford’s law–statistics on law enforcement’s repression efforts and on the reporting entities’ signalling efforts, published until and after the international evaluation.

*Group* ^*a*^	*N*	*χ*^2^	*KS*	*V*_N_*	*χ*^2^/*N*	*m*	d*	*Scatter*	$\bar{Staff}$
***Until the mutual evaluation***									
***1***	231	10.396	0.044	0.784	0.045	0.04	0.06	8	33.6
***2***	337	6.441	0.029	0.544	0.019	0.02	0.04	4	24.4
***3***	208	2.927	0.033	0.604	0.014	0.02	0.03	7	28.1
***4***	244	18.388**	0.053	1.72***	0.075	0.03	0.09	10	10.8
***After the mutual evaluation***									
**1**	158	3.86	0.051	0.917	0.02	0.02	0.04	5	42
**2**	552	27.87***	0.072***	2.11***	0.05	0.03	0.03	4	27.9
**3**	379	15.8**	0.066**	1.4**	0.04	0.04	0.04	4	32.8
**4**	212	22.22***	0.133***	2.02***	0.10	0.09	0.1	4	20.2

**Legend**

^**a**^ Group 1: BE, EE, NL, UK; Group 2: CY, CZ, ES, FI, HU, IE, MT, PT, LV; Group 3: AT, BG, DK, FR, IT, RO, SE, SL; Group 4: DE, EL, LT, LU, PL, SK. Staff represents the average staff of an FIU in the group, in that period.

*p< .1

**p< .05

***p< .01.

Finally, while the literature investigating strategic manipulation of national statistics has, generally, assumed a unitary decision maker that treats the country as a business (see Section 2), such assumption was not needed for the purposes of this paper. Numerous agencies participate in the national AML/CFT systems in Europe–*e*.*g*. financial sector, FIU, police, prosecution, judiciary etc. These agencies may vary in their preference for integer statistics and for a clean reputation. However, the conditions for them to manipulate statistics are met if (1) the integrity of statistics is not questioned by the agency itself or by its peers–hence, the opportunity to cheat exists–and if (2) manipulation is beneficial–thus, if negative evaluations are costly. As robustness check, I have re-run the analysis on three subsets: cross-border cash movements, repression, and suspicion reports, as these statistics originate from different institutions. Despite their differences, the patterns of deviations are similar (see Table D and Figure A in S2 File). The largest differences in deviations are explained by opportunities than by the source of the statistics. For instance, while repression data and data on suspicion reports deviated significantly more from Benford’s law after the mutual evaluation, data on cross-border cash movements deviated less after a mutual evaluation (see Table E in S2 File). Data on cross-border cash movements is registered on both sides of the border, and thereby, requires international coordination for strategic manipulation.

## 5. Discussion

Standard economic theory argues that cheating with official statistics is not possible [9]. Rational market players would be able to detect cheating and this would deter cheating from happening in the first place. Nevertheless, history shows that detection is often imperfect, and that strategic manipulation of official statistics happens. When Eurostat signalled that European statistics on compliance and efficiency in combatting money laundering do not entirely meet the standards of its Code of Practice, little exploration was conducted into the strategic nature of these statistics. The reasons were: (1) such an exercise was counter-intuitive, and (2) strategic manipulation was hard to prove. As a result, this puzzle remained practically unresolved and carried with it a possibly dangerous implication: sheltered by the impression that statistics accurately portray reality, the international community may increase pressure, with no real scope for success.

Next to needed, exploring the strategic nature of the European statistics on compliance and efficiency in combatting money laundering was also very intuitive, because of the framework in which combatting financial crime is organized. First, owing to the fact that official statistics are self-reported with various degrees of transparency towards the international community, opportunities for cheating were plentiful and varied over time. Second, countries faced a clear conflict of interest–either manipulate, invent or omit statistics–or exercise real efforts to combat money laundering nationally, thereby helping curb financial crime at a global level at the expense of the national business. Third, statistics on compliance and efficiency in combatting money laundering have been used to decide whether to impose economic sanctions. Consequently, this paper asked: *do countries consistently engage in misinforming the international community about their efforts to combat money laundering when faced with a conflict of interest and with the opportunities to do so*? The answer: *from the point of view of numerical anomalies*, *this hypothesis cannot be rejected*.

To answer this question, I have built a dataset of statistics that countries would have manipulated when pressured by the international community to show their performance in combatting money laundering. My dataset contained statistics for 27 EU Member States from 2003–2010 on, among others, the number of suspicion reports issued by national reporting entities, the number of investigations, prosecutions and convictions for money laundering undertaken by the national law enforcement agencies and the cross border cash movements overseen by the national agencies. I then used Benford’s law to identify the numerical anomalies that these statistics showed. First, I tested how well the usual explanations performed. Since they did not perform too well, I proceeded to compare the deviations from Benford’s law, of samples where strategic manipulation was more likely to occur, with samples where strategic manipulation was less likely to occur.

My analysis showed that statistics deviated less from Benford’s law, in the absence of opportunities–*e*.*g*. in the presence of peer monitoring, with increased transparency, and when statistics were recorded simultaneously across borders. Additionally, having a conflict of interest played a role. Consequently, the data showed that countries that were negatively evaluated by the international community exhibited significantly higher *ex-post* deviations from Benford’s law than countries whose statistics revealed an effective AML/CFT system. Statistics deviated less from Benford’s law when countries were monitored, and deviations could only be explained by the lack of FIU capacity only in the case of the FIUs that had very low staff numbers.

Importantly, my analysis revealed indications that EU statistics on compliance and efficiency in combatting money laundering are unreliable because Member States are evaluated on their basis. This is highly problematic, as it renders past statistics unreliable for the purpose of both policymaking and research. Moreover, if the framework for international evaluation remains unchanged, since the FATF standards have recently been readjusted for the purpose of the Fourth Round of Mutual Evaluations [78], new pressures may make future statistics even more unreliable.

Knowing this, what are some of the research venues to contemplate? I see several immediate possibilities: (1) applying Benford’s law to more recent and to future statistics on compliance and efficiency in combatting money laundering in the EU; (2) replicating this analysis on global datasets (*e*.*g*. statistics on money laundering from Asia-Pacific, the Americas and Africa); and (3) conducting interviews with AML/CFT officials who either retired or switched careers, and with non-governmental AML/CFT experts.

Given the ever-growing emphasis on the collection of statistics, I expect future datasets to allow for a richer application of Benford’s law. In spite of this, seeing how social sciences are reflexive sciences [79], it is likely that the efficiency of Benford’s law will erode with usage. Even so, there may be merit to applying Benford’s law to future statistics, if only as deterrent mechanism. Second, replicating the analysis on other regulatory regimes (e.g. Australia, US, Canada) is a straightforward way to test the hypotheses I put forward in this paper. While it does not guaranty the same results, it would answer the question: *“Is strategic manipulation of statistics on money laundering a European problem*, *or a natural consequence of the compliance design of the FATF’s strategy*?” Finally, conducting interviews with non-active governmental officials and with AML/CFT experts may expose potential suspicions of manipulation and even discussions about the negative consequences arising from faithful reporting. If the analysis I presented in this paper is correct, and if interviewees do not have high implicit bias or significant disclosure barriers, a significant number of them should be knowledgeable about at least some aspects of this strategic manipulation.

## Supporting Information

S1 File Data description. (DOCX)

S2 File Robustness checks. (DOCX)
