# Supplementary material for: Do Countries Consistently Engage in Misinforming the International Community about Their Efforts to Combat Money Laundering? Evidence Using Benford’s Law
Source: PLoS One. 2017 Jan 25;12(1):e0169632. doi: 10.1371/journal.pone.0169632 (PMC5266253; doi:10.1371/journal.pone.0169632)
Supplement: S1 File — (DOCX) [file pone.0169632.s001.docx]

**S1 File. Data description**

Table A reports on the availability of statistics on compliance and efficiency in combatting money laundering across the EU-27, from 2003 to 2010, excluding the null statistics. Although national samples are sufficiently scattered, some countries (*a.o.* Greece, Ireland, Poland and Slovakia) have too few observations to allow for the application of Benford’s law while distinguishing between countries.

**Table A. Number of available statistics on compliance and efficiency in combatting money laundering (by year) and degree of scatter, per EU Member State.**

|  | **#Obs in 2003** | **#Obs in 2004** | **#Obs in 2005** | **#Obs in 2006** | **#Obs in 2007** | **#Obs in 2008** | **#Obs in 2009** | **#Obs in 2010** | **Total # Obs** | **Scatter** |
| --- | --- | --- | --- | --- | --- | --- | --- | --- | --- | --- |
| **AT** | 2 | 11 | 13 | 11 | 14 | 21 | 21 | 23 | **116** | **9** |
| **BE^X^** |  |  | 3 | 6 | 7 | 26 | 25 | 25 | **92** | **7** |
| **BG** | 13 | 17 | 13 | 13 | 17 | 23 | 20 | 22 | **138** | **8** |
| **CY** | 10 | 10 | 12 | 11 | 14 | 20 | 17 | 22 | **116** | **7** |
| **CZ^X^** | 10 | 10 | 11 | 11 | 11 | 15 | 16 | 17 | **101** | **8** |
| **DE** | 12 | 12 | 16 | 16 | 16 | 23 | 25 | 22 | **142** | **10** |
| **DK^X^** | 7 | 11 | 7 | 11 | 12 | 19 | 18 | 18 | **103** | **7** |
| **EE** | 7 | 9 | 13 | 15 | 20 | 26 | 26 | 23 | **139** | **8** |
| **EL^X^** | 6 | 6 | 6 | 6 | 6 | 16 | 12 | 11 | **69** | **7** |
| **ES** | 15 | 16 | 16 | 16 | 16 | 22 | 24 | 15 | **140** | **8** |
| **FI** | 14 | 17 | 18 | 18 | 20 | 27 | 26 | 26 | **166** | **7** |
| **FR^X^** | 4 | 7 | 8 | 9 | 9 | 25 | 25 | 15 | **102** | **9** |
| **HU** | 6 | 8 | 12 | 13 | 13 | 25 | 25 | 24 | **126** | **7** |
| **IE^X^** | 10 | 11 | 11 | 13 | 13 | 8 | 8 | 8 | **82** | **6** |
| **IT** | 8 | 8 | 10 | 16 | 17 | 22 | 24 | 25 | **130** | **9** |
| **LT^X^** | 4 | 9 | 10 | 10 | 12 | 17 | 16 | 19 | **97** | **8** |
| **LU** | 9 | 12 | 12 | 16 | 14 | 19 | 23 | 22 | **127** | **6** |
| **LV** | 12 | 13 | 15 | 18 | 19 | 22 | 23 | 19 | **141** | **7** |
| **MT** | 14 | 9 | 12 | 13 | 17 | 20 | 22 | 19 | **126** | **7** |
| **NL** | 14 | 15 | 14 | 16 | 16 | 21 | 21 | 21 | **138** | **8** |
| **PL^X^** | 1 | 4 | 8 | 8 | 12 | 20 | 13 | 9 | **75** | **8** |
| **PT** | 7 | 6 | 9 | 11 | 13 | 23 | 22 | 22 | **113** | **8** |
| **RO** | 3 | 5 | 14 | 17 | 20 | 25 | 25 | 24 | **133** | **8** |
| **SE** | 6 | 8 | 8 | 11 | 13 | 22 | 22 | 22 | **112** | **8** |
| **SK^X^** | 1 | 1 | 3 | 7 | 13 | 18 | 19 | 20 | **82** | **6** |
| **SL^X^** | 7 | 8 | 9 | 10 | 10 | 19 | 19 | 19 | **101** | **9** |
| **UK** | 14 | 14 | 14 | 14 | 0 | 18 | 21 | 21 | **115** | **9** |
| **EU-27** | **216** | **257** | **297** | **336** | **364** | **562** | **558** | **533** | **3122** | **9** |

***Notes.*** ^X^ too few observations to correctly apply Benford’s law.

Recommendation 13 states that financial organizations should report any transactions or activity where they suspect money laundering to the FIU [57]. Recommendation 32 states that countries should review their effectiveness by maintaining accurate statistics [57]. Falling under Recommendations 13, 16 and 32, my dataset includes the number of suspicion reports forwarded by credit institutes, life insurance companies, investment firms, money transfer agencies, exchange offices, lawyers, notaries, real estate agents, traders in goods of value above Euros 15,000, casinos, external accountants or auditors and other obliged entities [1,76]. Falling under Recommendation 32 alone, I collected data on the number of cases brought to prosecution originating from suspicion reports, cash transaction reports and independent law enforcement investigations, the total number of persons or legal entities convicted for money laundering offences, and the number of sentences for money laundering offences [1,76]. Further, recommendation 27 states that designated law enforcement authorities should be made responsible for investigating money laundering, and that for this purpose they should make use of the available special investigative techniques [57]. Falling under Recommendations 27 and 32, I collected data on the number of postponement orders adopted on reported transactions [76] and on the number of money laundering investigations carried out independently by law enforcement agencies, without a prior suspicion report [76]. Recommendation 31 states that policy makers, FIUs, supervisors and law enforcement should cooperate and coordinate in order to effectively combat money laundering [57]. Falling under Recommendations 31 and 32, I gathered data on the number of suspicion reports sent by the FIU to the law enforcement which are thereafter investigated; and on the number of cases initiated by law enforcement agencies on the basis of suspicion reports sent by the FIU [1,76]. Finally, special recommendation IX states that countries should be able to detect the physical cross-border transportation of currency and bearer negotiable instruments and to stop those that are suspicious and to sanction those who do not truthfully report on their cash cross-border transports [58]]. Falling under the Special Recommendation IX, I collected data on the number of declarations made in application to the EU Cash Control Regulation on entering the EU and on leaving the EU, on the amounts in millions of Euros that these declarations contained, on the number of incorrect cash declarations or findings as a result of customs controls in the EU at external borders - on entering and on leaving the EU, on the amounts in millions of Euros that these incorrect declarations contained, and on the number of suspicious cash activities at the EU borders that are reported to the FIU [1,76].

Table B offers descriptive statistics on the data. Table B shows that only a few variables have sufficient observations and are sufficiently scattered, to allow for a correct application of Benford’s law. Applying Benford’s law to those variables that are not sufficiently scattered or too small, Benford’s law would be rejected by design. I therefore cannot conclude anything about variable specific variations using this dataset. For the same reason, as Table A shows, I have refrained from using Benford’s law to compare deviations across EU member states.

**Table B. Number of observations per indicator of compliance and efficiency in combatting money laundering (by year), and degree of scatter, pooled across the EU.**

|  | **#Obs in 2003** | **#Obs in 2004** | **#Obs in 2005** | **#Obs in 2006** | **#Obs in 2007** | **#Obs in 2008** | **#Obs in 2009** | **#Obs in 2010** | **Total # Obs** | **Scatter** |
| --- | --- | --- | --- | --- | --- | --- | --- | --- | --- | --- |
| **Repression statistics** | | | | | | | | | | |
| **#Postponement orders ^X^** | 8 | 11 | 14 | 15 | 17 | 17 | 19 | 17 | **117** | **3** |
| **#Border reports ^X^** | 7 | 9 | 8 | 10 | 15 | 17 | 15 | 16 | **97** | **3** |
| **#ML investigations without SR ^X^** | 6 | 9 | 11 | 14 | 15 | 14 | 14 | 15 | **98** | **3** |
| **#SR analysed by Law Enforcement** | 9 | 12 | 16 | 18 | 20 | 19 | 18 | 18 | **130** | **4** |
| **#Law Enforcement cases from SR** | 13 | 14 | 15 | 19 | 20 | 18 | 12 | 13 | **124** | **4** |
| **#ML prosecutions** | 8 | 12 | 13 | 19 | 22 | 23 | 18 | 17 | **132** | **4** |
| **#Persons convicted ^X^** | 9 | 12 | 15 | 19 | 20 | 22 | 20 | 16 | **133** | **3** |
| **#ML sentences ^X^** | 11 | 13 | 16 | 19 | 19 | 16 | 17 | 14 | **125** | **3** |
| **Cross border cash movements statistics** | | | | | | | | | | |
| **#Incorrect cash declarations entering the EU ^X^** |  |  |  |  |  | 22 | 21 | 24 | **67** | **3** |
| **#Incorrect cash declarations on leaving the EU ^X^** |  |  |  |  |  | 19 | 25 | 21 | **65** | **3** |
| **Total # of incorrect cash declarations ^X^** |  |  |  |  |  | 25 | 25 | 24 | **74** | **3** |
| **Amount of cash incorrectly declared ^X^** |  |  |  |  |  | 23 | 25 | 24 | **72** | **4** |
| **#Cash declarations on entering the EU ^X^** |  |  |  |  |  | 27 | 27 | 27 | **81** | **4** |
| **#Cash declarations on leaving the EU ^X^** |  |  |  |  |  | 26 | 26 | 26 | **78** | **4** |
| **Total # of cash declarations** | 6 | 6 | 6 | 7 | 18 | 27 | 27 | 27 | **124** | **4** |
| **Amount of cash declared at borders ^X^** |  |  |  |  | 6 | 27 | 27 | 27 | **87** | **5** |
| **Suspicion reports statistics** | | | | | | | | | | |
| **#SR from investment firms ^X^** | 9 | 7 | 12 | 9 | 12 | 16 | 13 | 8 | **86** | **3** |
| **#SR from exchange offices ^X^** | 9 | 8 | 8 | 10 | 10 | 10 | 9 | 9 | **73** | **4** |
| **#SR from notaries ^X^** | 8 | 9 | 7 | 9 | 10 | 14 | 16 | 14 | **87** | **2** |
| **#SR from accountants & auditors ^X^** | 7 | 7 | 10 | 11 | 13 | 16 | 16 | 13 | **93** | **4** |
| **#SR from casinos ^X^** | 7 | 12 | 11 | 12 | 11 | 14 | 16 | 16 | **99** | **3** |
| **#SR from traders high value goods ^X^** | 4 | 4 | 6 | 10 | 7 | 11 | 11 | 9 | **62** | **3** |
| **#SR from lawyers ^X^** | 6 | 10 | 15 | 14 | 17 | 19 | 18 | 17 | **116** | **3** |
| **#SR from real estate agents ^X^** | 3 | 6 | 9 | 12 | 12 | 12 | 13 | 14 | **81** | **2** |
| **#SR from life insurance companies ^X^** | 11 | 13 | 18 | 17 | 15 | 16 | 16 | 19 | **125** | **3** |
| **#SR from credit organizations** | 20 | 22 | 24 | 25 | 24 | 26 | 26 | 24 | **191** | **4** |
| **#SR from money transfer companies** | 14 | 15 | 16 | 19 | 16 | 16 | 21 | 19 | **136** | **5** |
| **#SR from other obliged entities** | 17 | 20 | 21 | 22 | 20 | 24 | 22 | 20 | **166** | **5** |
| **Total no of SR** | 24 | 26 | 26 | 26 | 25 | 26 | 25 | 25 | **203** | **4** |

***Notes.*** ^X^ too few observations to correctly apply Benford’s law.

The FIU is pivotal to the AML/CFT strategy of the FATF. Table C reports on the capacity statistics of the European FIUs [1,76].

**Table C. Number of staff dedicated full time (or full time equivalent) to money laundering in the FIU**

| **Country** | **2003** | **2004** | **2005** | **2006** | **2007** | **2008** | **2009** | **2010** |
| --- | --- | --- | --- | --- | --- | --- | --- | --- |
| **AT** |  |  |  |  |  |  |  |  |
| **BE** |  |  |  |  |  |  |  | 51 |
| **BG** | 34 | 34 | 36 | 44 | 42 | 41 |  |  |
| **CY** | 14 | 14 | 14 | 19 | 19 | 19 | 21 | 21 |
| **CZ** | 28 | 27 | 25 | 27 | 27 | 15 | 15 | 19 |
| **DK** |  | 10 | 11 | 12 | 14 | 18 | 18 | 14 |
| **EE** | 7 | 9 | 11 | 12 | 17 | 22 | 18 | 16 |
| **FI** | 27 | 27 | 27 | 27 | 27 | 27 | 25 | 24 |
| **FR** |  |  | 37 | 48 | 47 | 46 | 51 |  |
| **DE** | 14 | 16 | 16 | 16 | 16 | 17 | 17 | 19 |
| **EL** | 3 | 3 | 3 | 4 | 15 | 18 |  |  |
| **HU** |  |  |  |  |  |  | 31 | 30 |
| **IE** | 7 | 7 | 7 | 7 | 8 |  |  |  |
| **IT** |  |  |  |  | 92 | 99 | 97 | 104 |
| **LV** | 17 | 18 | 19 | 18 | 19 | 19 | 19 | 19 |
| **LT** | 9 | 10 | 12 | 12 | 12 | 13 | 13 | 13 |
| **LU** | 4 | 4 | 5 | 5 | 6 | 6 | 6 | 6 |
| **MT** | 3 | 3 | 3 | 3 | 6 | 6 | 8 | 10 |
| **NL** |  |  |  | 56 | 56 | 56 | 56 | 56 |
| **PL** |  |  |  |  |  |  |  |  |
| **PT** | 29 | 26 | 25 | 26 | 27 | 27 | 27 | 28 |
| **RO** | 39 | 41 | 39 | 27 | 35 | 34 | 36 | 36 |
| **SK** |  | 29 | 29 | 29 | 29 | 28 | 38 | 38 |
| **SL** | 13 | 15 | 15 | 14 | 15 | 16 | 18 | 18 |
| **ES** | 79 | 75 | 80 | 77 | 79 | 78 | 77 | 80 |
| **SE** |  | 15 | 15 | 15 | 15 | 17 | 14 | 14 |
| **UK** |  |  |  |  |  | 103 |  |  |

***Source.*** The 2010 and the 2013 EUROSTAT reports on Money Laundering in Europe.
