# Supplementary material for: Do Countries Consistently Engage in Misinforming the International Community about Their Efforts to Combat Money Laundering? Evidence Using Benford’s Law
Source: PLoS One. 2017 Jan 25;12(1):e0169632. doi: 10.1371/journal.pone.0169632 (PMC5266253; doi:10.1371/journal.pone.0169632)
Supplement: S2 File — (DOCX) [file pone.0169632.s002.docx]

**S2 File. Robustness checks**

Table D reports on the conformity to Benford’s law given the temporal distance to the international evaluation, of three sub-samples: cross border cash movements, the repression and suspicion reports. First, data on cross border cash movements is too scarce to draw any conclusions on the fact that deviations from Benford’s law decrease over time. Statistics on the repression of money laundering are also scarce and suffice for the application of Benford’s law only to the second evaluation cycle. Table D shows that statistics on repression of money laundering were higher the year before the writing of the FUR than the year when the FUR was written. Finally, statistics on suspicion reports were sufficiently abundant. Also, these statistics deviated significantly in the absence of international monitoring.

**Table D. Robustness check: assessment of conformity to Benford’s law before and during evaluations of three subsamples: cross border cash movements, repression, and suspicion reports statistics.**

| ***Variables*** | $\boldsymbol{N}$ | $\boldsymbol{\chi}^{\boldsymbol{2}}$ | $\boldsymbol{KS}$ | ${\boldsymbol{V}_{\boldsymbol{N}}}^{\boldsymbol{*}}$ | $\boldsymbol{\chi}^{\boldsymbol{2}}\boldsymbol{/}\boldsymbol{N}$ | $\boldsymbol{m}$ | $\boldsymbol{d}^{\boldsymbol{*}}$ | ***Scatter*** |
| --- | --- | --- | --- | --- | --- | --- | --- | --- |
| ***Cash stats at MER-2 ^X^*** | *23* | *6.69* | *0.177* | *0.91* | *0.29* | *0.18* | *0.26* | 9 |
| ***Cash stats at MER-1 ^X^*** | *35* | *3.35* | *0.066* | *0.45* | *0.10* | *0.04* | *0.09* | 10 |
| ***Cash stats at MER ^X^*** | *50* | *4.5* | *0.09* | *0.78* | *0.09* | *0.06* | *0.10* | 10 |
| ***Cash stats at FUR-1 ^X^*** | *55* | *10.23* | *0.1* | *0.82* | *0.19* | *0.06* | *0.11* | 10 |
| ***Cash stats at FUR ^X^*** | *81* | *8.19* | *0.126** | *1.22** | *0.10* | *0.06* | *0.10* | 9 |
| ***Cash stats at FUR+1*** | *140* | *3.64* | *0.066* | *0.84* | *0.03* | *0.05* | *0.06* | 9 |
| ***Repressive stats at MER-3 ^X^*** | *53* | *4.02* | *0.133* | *1.43** | *0.076* | *0.13* | *0.14* | 4 |
| ***Repressive stats at MER-2 ^X^*** | *82* | *9.62* | *0.099* | *1.06* | *0.117* | *0.07* | *0.11* | 4 |
| ***Repressive stats at MER-1 ^X^*** | *102* | *10.85* | *0.065* | *0.84* | *0.106* | *0.06* | *0.10* | 4 |
| ***Repressive stats at MER*** | *112* | *18.05*** | *0.092* | *1* | *0.161* | *0.08* | *0.10* | 4 |
| ***Repressive stats at FUR-1*** | *122* | *12.62* | *0.125**** | *1.5*** | *0.103* | *0.13* | *0.14* | 4 |
| ***Repressive stats at FUR*** | *126* | *9.26* | *0.048* | *0.56* | *0.073* | *0.05* | *0.09* | 4 |
| ***Repressive stats at FUR+1 ^X^*** | *116* | *15.27** | *0.069* | *0.84* | *0.132* | *0.07* | *0.11* | 3 |
| ***SR stats at MER-3 ^X^*** | 103 | 14.5* | 0.097 | 1 | 0.141 | 0.1 | 0.14 | 4 |
| ***SR stats at MER-2*** | 159 | 8.67 | 0.076 | 1.29* | 0.055 | 0.06 | 0.07 | 5 |
| ***SR stats at MER-1*** | 181 | 10.02 | 0.041 | 0.63 | 0.055 | 0.03 | 0.05 | 5 |
| ***SR stats at MER*** | 187 | 8.45 | 0.068 | 1 | 0.045 | 0.05 | 0.06 | 5 |
| ***SR stats at FUR-1*** | 149 | 13.13* | 0.095** | 1.19* | 0.088 | 0.08 | 0.1 | 4 |
| ***SR stats at FUR*** | 167 | 9.75 | 0.08* | 1.27* | 0.058 | 0.04 | 0.06 | 5 |
| ***SR stats at FUR+1*** | 153 | 15.53** | 0.137*** | 2.45*** | 0.102 | 0.06 | 0.09 | 5 |

**Notes.** ^a^ Stats at MER-3, ML stats at MER-2, ML stats at MER-1 and ML stats at MER aggregate statistics on compliance and efficiency in combatting money laundering that were published 3,2 and 1 year before and respectively during the year of the international evaluation. Stats at FUR-1 and ML stats at FUR aggregate the statistics published the year before and respectively during the compilation of the FUR. ML stats at FUR+1 aggregate statistics on compliance and efficiency in combatting money laundering that were published the year the FUR was published and discussed in the plenary. ^X^ too few observations or degrees of scatter to correctly apply Benford’s law. *p< .1, **p< .05, ***p< .01.

Fig A plots data in Table D. Spikes in deviations occurred at the beginning of the assessment cycles and by the end of an assessment cycle, deviations were smallest. The five measures of deviation followed similar patters especially on sub-samples that were sufficiently scattered and large.


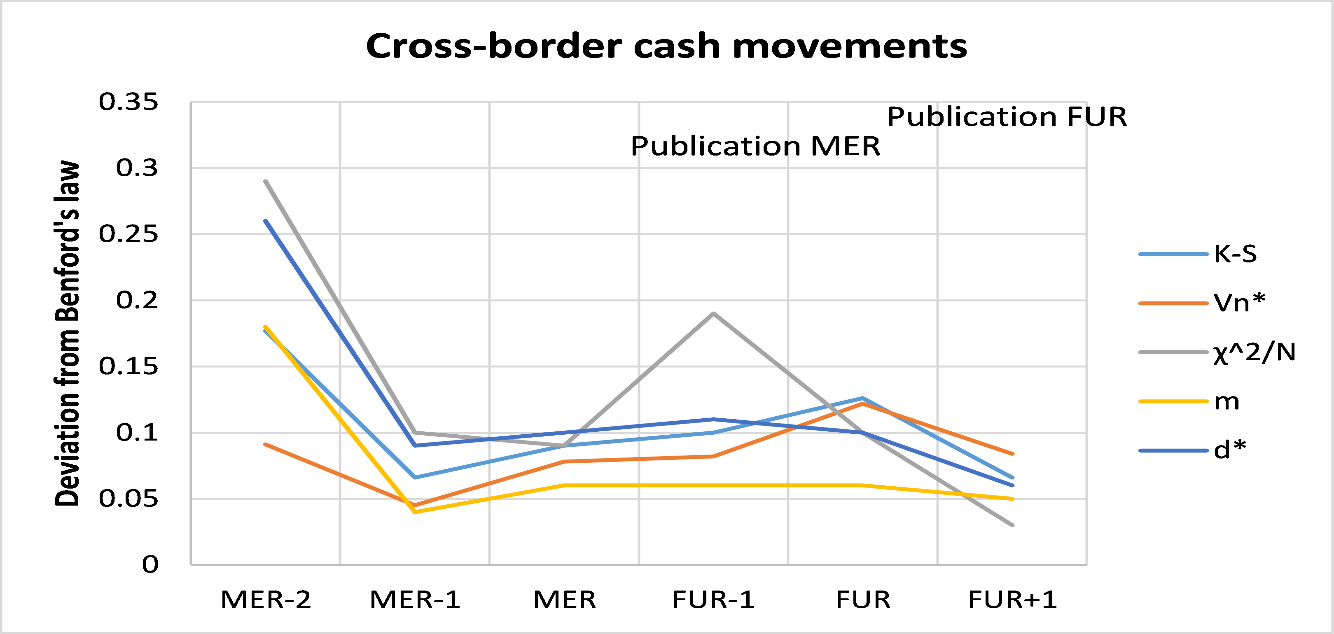

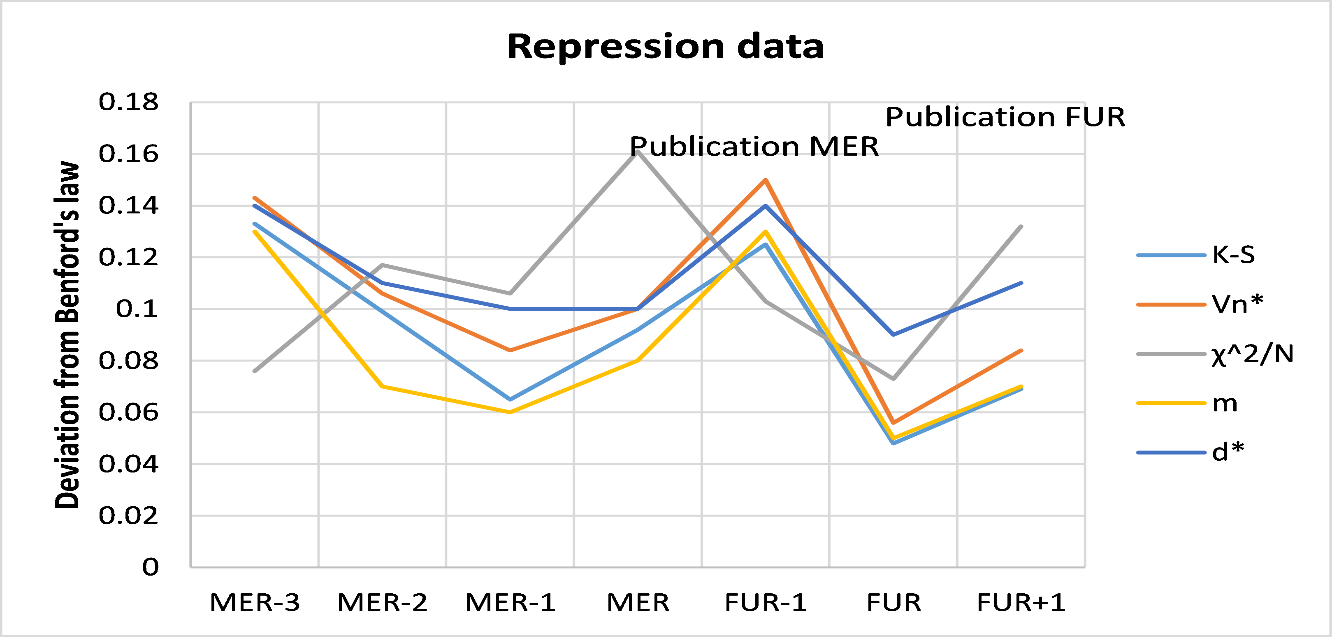

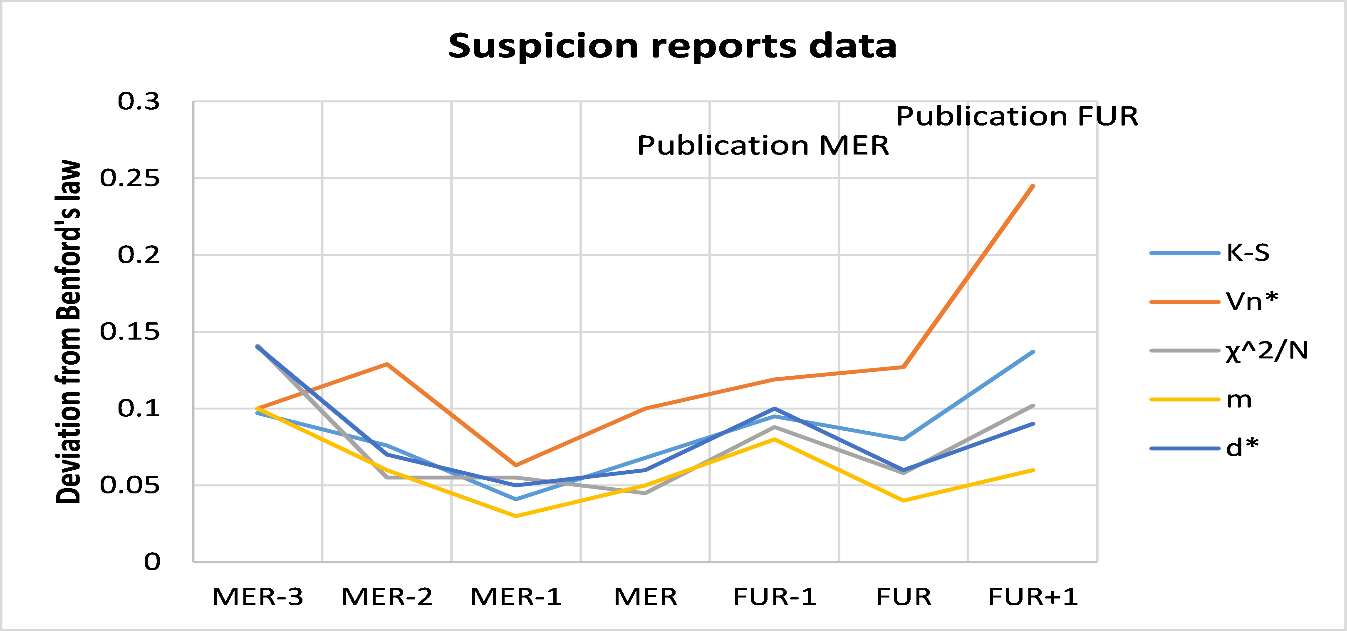


**Fig A. Robustness check: timely assessment of conformity to Benford’s law of three subsamples: cross border cash movements, repression, and suspicion reports statistics. While in accordance with Table D, for visual purposes the measures**${\mathbf{V}_{\mathbf{N}}}^{\mathbf{*}}$ **has been divided by a factor 10.**

Table E owreports on the conformity to Benford’s law of aggregate statistics and of the three subsamples before and since the MER was published: data on the effort of law enforcement/ the repression statistics, data on cash cross-border movements, and data on suspicion reporting. Samples were composed such that they were both sufficiently large and scattered to allow for the application of Benford’s law. Table E shows that statistics on cross-border cash movements deviated less after the MER was published. Statistics on repression of money laundering deviated relatively little before the MER was published but strongly and significantly after. Similarly, statistics on suspicion reports deviated more after the MER was published.

**Table E. Robustness check: assessment of conformity to Benford’s law (before and after the publication of the MER) of the aggregate statistics and of three subsamples: repression, cross border cash movements and suspicion reports statistics.**

| ***Variables*** | $\boldsymbol{N}$ | $\boldsymbol{\chi}^{\boldsymbol{2}}$ | $\boldsymbol{KS}$ | ${\boldsymbol{V}_{\boldsymbol{N}}}^{\boldsymbol{*}}$ | $\boldsymbol{\chi}^{\boldsymbol{2}}\boldsymbol{/}\boldsymbol{N}$ | $\boldsymbol{m}$ | $\boldsymbol{d}^{\boldsymbol{*}}$ | ***Scatter*** |
| --- | --- | --- | --- | --- | --- | --- | --- | --- |
| ***ML stats before MERp*** | 1281 | 12.42 | 0.024 | 0.86 | 0.01 | 0.01 | 0.01 | 10 |
| ***ML stats since MERp*** | 1873 | 34.81*** | 0.056*** | 2.73*** | 0.02 | 0.01 | 0.01 | 10 |
| ***Repression stats before MERp*** | 406 | 8.44 | 0.039 | 0.89 | 0.02 | 0.01 | 0.02 | 4 |
| ***Repression stats since MERp*** | 582 | 39.01*** | 0.099*** | 2.54*** | 0.07 | 0.04 | 0.05 | 4 |
| ***Cross-border cash stats before MERp*** | 112 | 10.79 | 0.056 | 0.63 | 0.10 | 0.05 | 0.09 | 10 |
| ***Cross-border cash stats since MERp*** | 536 | 8.55 | 0.048 | 1.29** | 0.02 | 0.01 | 0.02 | 10 |
| ***Suspicion reports stats before MERp*** | 763 | 11.09 | 0.044** | 1.22* | 0.01 | 0.02 | 0.02 | 5 |
| ***Suspicion reports stats since MERp*** | 755 | 17.03** | 0.069*** | 2.45*** | 0.02 | 0.02 | 0.02 | 5 |

***Notes.*** Statistics marked ‘before MERp’ aggregate data from 2003 until the year before the MER was published; statistics marker ‘since MERp’ aggregate data from the moment the evaluation is published until 2010.*p< .1, **p< .05, ***p< .01.
